# Primary leiomyosarcoma of the spine: A case report and literature review

**DOI:** 10.1097/MD.0000000000006227

**Published:** 2017-03-03

**Authors:** Yi Yang, Litai Ma, Lingli Li, Hao Liu

**Affiliations:** Department of Orthopedics, West China Hospital, Sichuan University, Chengdu, Sichuan Province, China.

**Keywords:** diagnosis, literature review, Primary leiomyosarcoma, spine, vertebra

## Abstract

**Rationale:**

Primary leiomyosarcoma of the bone was firstly reported by Evans and Sanerkin in 1965, whereas primary leiomyosarcoma of the vertebra is extremely rare. Because of the rarity of primary vertebral leiomyosarcoma, the diagnosis, treatment, and clinical outcome still remain controversial. Here we report a special case of primary leiomyosarcoma in the thoracic vertebra.

**Patient concerns:**

A 47-year-old female patient was admitted to our institution with the chief complaint of persistent back pain for 4 weeks. She had no symptoms of numbness, weakness, pain, and abnormal sensation in her extremities.

**Diagnoses:**

Neurological examination on admission revealed no obvious abnormality. Magnetic resonance imaging showed a bone destruction of the T11 vertebral body and the right pedicle. Therefore, primary vertebral leiomyosarcoma was suspected. Pathological hematoxylin and eosin staining of the resected tumor revealed a diagnosis of polymorphic undifferentiated sarcoma. Furthermore, to identify the subtype of this sarcoma, the immunohistochemical staining of the tumor was performed with each of the various antibodies and the results are epithelial membrane antigen (−), H-caldesmon (−), desmin (+), smooth muscle actin (+), S-100 (−), myogenin (−), pan-keratin (−), and Ki-67 (positive rate: 20%). Finally, the patient was diagnosed as primary vertebral leiomyosarcoma.

**Interventions:**

the anterior corpectomy and autogenous iliac bone graft with instrumentation combined with the posterior spinal canal decompression and fusion with the pedicle screw system were performed through an anterior-posterior union approach.

**Outcomes:**

Neither clinical symptoms nor signs of tumor recurrence were detected within the follow-up of 6 months. In addition, 11 cases of the primary vertebral leiomyosarcoma reported in the literature were reviewed and summarized.

**Lessons:**

Exclusion of metastatic leiomyosarcoma by various imaging modalities and histopathological examinations, especially the immunohistochemical staining with various antibodies against the epithelial and mesenchymal cell markers, are critical for establishing the correct diagnosis of the primary vertebral leiomyosarcoma. Surgical resection, especially the total en bloc spondylectomy, is the main treatment option with a good outcome, albeit with a limited follow-up duration.

## 1 Introduction

Leiomyosarcoma originates from smooth muscle cells and usually occurs in the retroperitoneum and abdomen.^[1]^ Primary leiomyosarcoma of the bone is very rare and was firstly reported by Evans and Sanerkin in 1965^[2]^ and it most commonly involves the bones around the knee.^[3]^ Primary leiomyosarcoma of the spinal vertebra is extremely rare. In fact, it is difficult to make a diagnosis of primary spinal leiomyosarcoma because it needs to exclude the possibility of a metastatic tumor to the spinal bone and the clinical and radiological findings of the spinal vertebrae resulted from the primary vertebral leiomyosarcoma are generally nonspecific. However, the immunohistochemical staining has been playing an increasingly more important role in the diagnosis of leiomyosarcoma in recent years.

Surgery is an effective method for the treatment of primary spinal vertebral tumors. The total en bloc spondylectomy has been introduced in recent years with an advantage of minimizing the local recurrence.^[4]^ Chemotherapy, radiotherapy, and biotherapy are also the options of treatment for primary spinal tumors. However, because of its rarity, the diagnosis, treatment, and clinical outcome of the primary spinal vertebral leiomyosarcoma still remain controversial.

We here report a special case of primary leiomyosarcoma in the thoracic vertebra, T11. The patient underwent an anterior-posterior union approach surgery for tumor resection, combined with an autogenous iliac bone graft and instrumentation. In addition, the primary spinal vertebral leiomyosarcoma cases previously reported in the literature were reviewed and the characteristics of diagnosis, treatment, and clinical outcomes of these cases were summarized.

## 2 Case report

**Figure 1:**
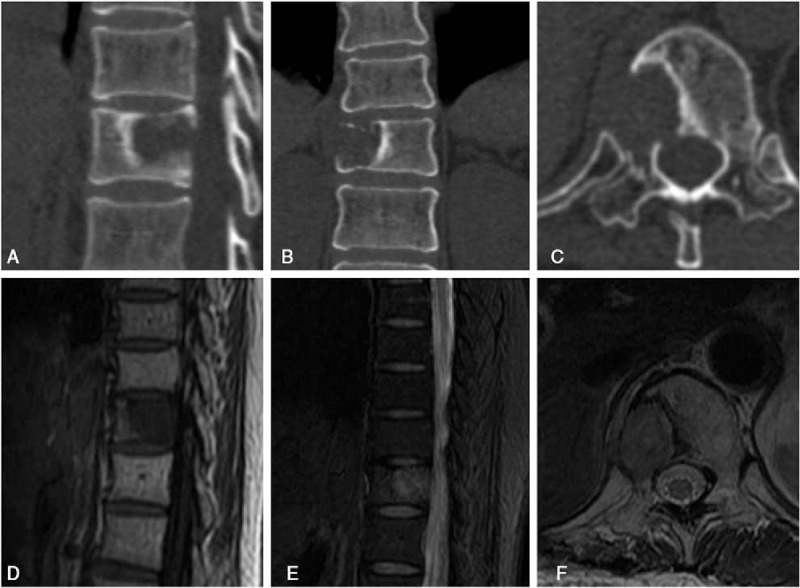
Preoperative 3-dimensional computed tomography (CT) scan image and magnetic resonance imaging (MRI). (A) Sagittal CT; (B) coronal CT; (C) axial CT; (D) T1 sagittal MRI; (E) T2 sagittal MRI; (F) axial MRI.

**Figure 2:**
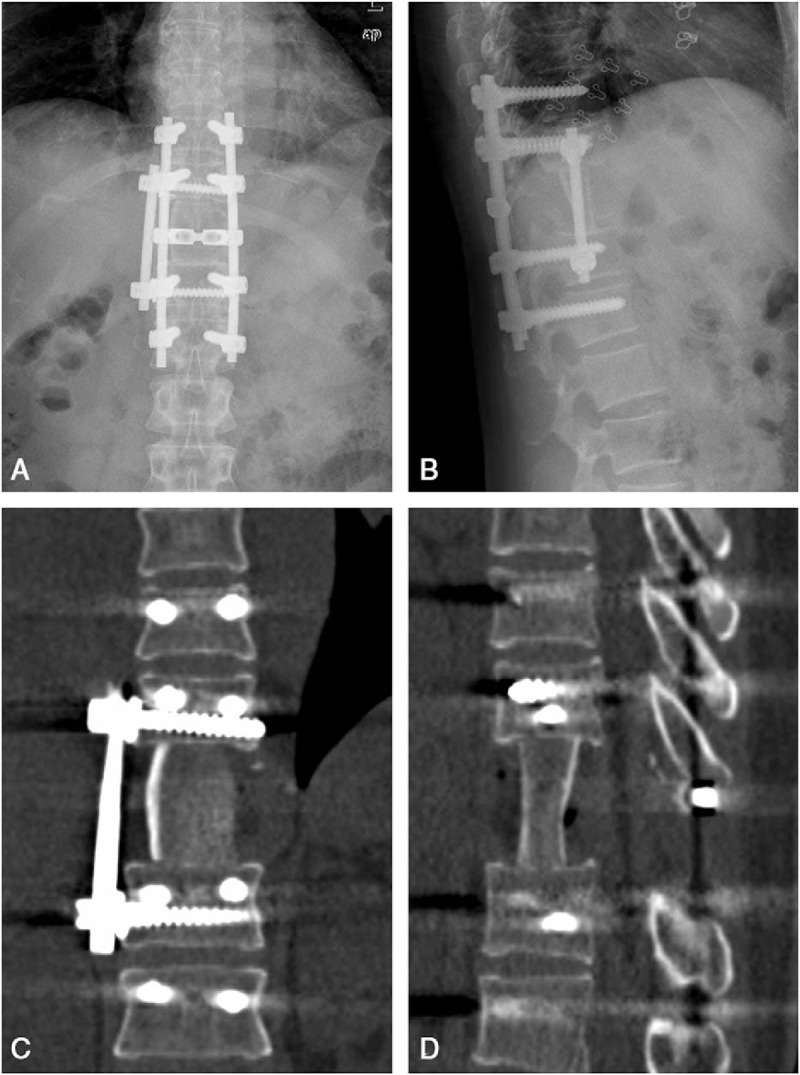
Postoperative immediate x-ray and 3-dimensional computed tomography (CT) scan images. (A) Anteroposterior x-ray; (B) Lateral x-ray; (C) coronal CT; (D) Sagittal CT.

**Figure 3:**
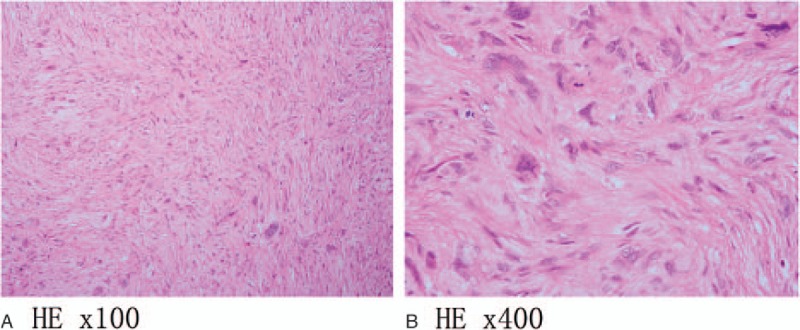
Photomicrographs of pathological hematoxylin and eosin staining of the tissue sections of the resected primary vertebral leiomyosarcoma. These photomicrographs (A and B) show a large number of hyperplastic collagen fibers and heteromorphic cells that support a diagnosis of polymorphic undifferentiated sarcoma.

**Figure 4:**
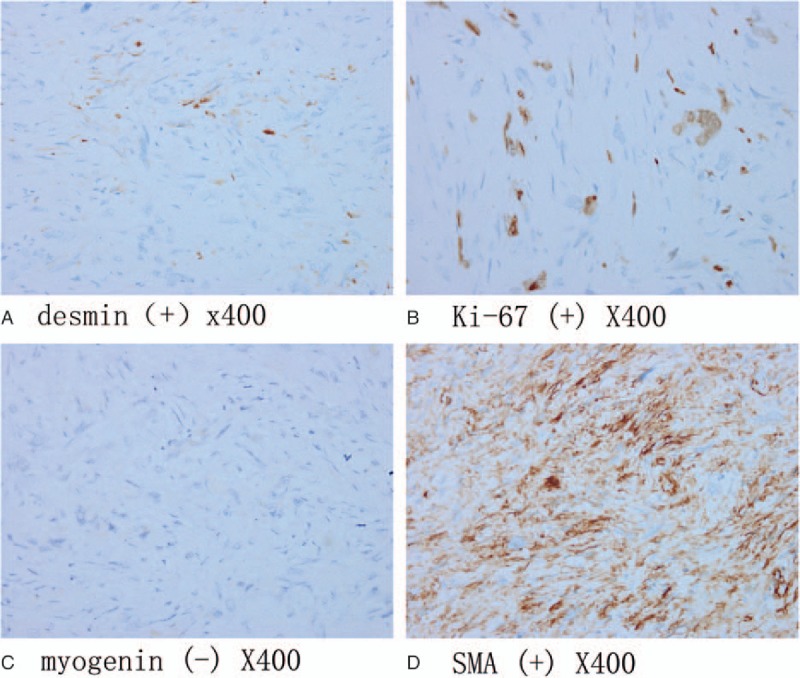
The representative photomicrographs of immunohistochemical staining of the tissue sections of the resected primary vertebral leiomyosarcoma. (A) Desmin (+), (B) Ki-67 (positive rate: 20%), (C) myogenin (−), and (D) smooth muscle actin (+).

**Figure 5:**
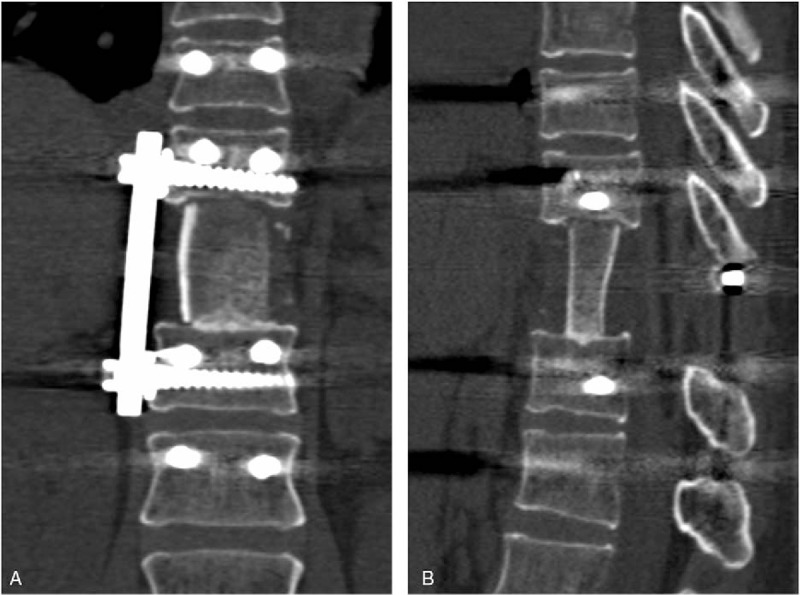
Postoperative 3-dimensional computed tomography (CT) scan images in the 6 months. (A) Coronal CT; (B) sagittal CT.

A 47-year-old female patient was admitted to our institution with the chief complaint of persistent back pain for 4 weeks. Her pain aggravated at night and could not be relieved by conventional analgesics. She experienced no symptoms of numbness, weakness, pain, and abnormal sensation in her extremities. Neurological examination on admission revealed no obvious abnormality. She had normal myodynamia of the 4 limbs, normal sensation of the skin, Hoffmann sign (−), and Babinski sign (−). She had no history of tumor, allergy, and surgeries. Laboratory findings were within the normal limits except for the following: erythrocyte sedimentation rate 46.0 mm/h, red blood cell 3.57 × 10^12^ cells/L, hemoglobin 105 g/L, and albumin 38.0 g/L. Tumor markers including alpha fetoprotein, carcinoembryonic antigen (CEA), carbohydrate antigen 15–3 (CA15–3), carbohydrate antigen-125 , carbohydrate antigen19–9 (CA19–9), carbohydrate antigen 72–4 (CA72–4), cytokeratin fragment antigen 21–1 (CYFRA21–1), and neuron-specific enolase were all within the normal limits while bone specific alkaline phosphatase was 7.59 μg/L. Three-dimensional computed tomography (CT) scan and magnetic resonance imaging (MRI) showed a bone destruction of the T11 vertebral body and the right pedicle, but the endorachis and medulla spinalis were not destructed (Fig. 1). Chest x-ray, abdominal ultrasonography, and other examinations showed no significant abnormity. The positron emission tomography/computed tomography (PET/CT) showed a tumor lesion at the T11 and no other tumor-like findings in the rest of her body. The patient was preliminarily diagnosed as an unknown primary tumor of spinal vertebra. Anterior corpectomy and an autogenous iliac bone graft with instrumentation combined with the posterior spinal canal decompression and fusion with the pedicle screw system were performed through an anterior-posterior union approach (Fig. 2). The resected tumor was grossly grayish-white, elastically hard, and with a sand-like appearance. The surgically resected tissue was sent to the department of pathology in our institution for histopathological examination. The hematoxylin and eosin (HE) staining and immunohistochemical staining with various antibodies against the epithelial and mesenchymal cell markers were performed to determine the identity of the tumor. To our surprise, the HE staining showed a large number of hyperplastic collagen fibers and heteromorphic cells that supported a diagnosis of polymorphic undifferentiated sarcoma (Fig. 3). The results of the immunohistochemical staining were epithelial membrane antigen −) (data not shown), H-caldesmon (−) (data not shown), desmin (+) (Fig. 4A), Ki-67 (positive rate: 20%) (Fig. 4B), myogenin (−) (Fig. 4C), smooth muscle actin (SMA) (+) (Fig. 4D), S-100 (−) (data not shown), and pan-keratin (−) (data not shown). Finally the patient was diagnosed as having primary leiomyosarcoma of spinal vertebra. The symptom of back pain was relieved gradually and the patient was discharged 7 days after surgery without any postoperative therapy. Within the follow-up of 6 months, no clinical symptoms and signs of tumor recurrence were detected (Fig. 5).

## 3 Discussion

Exclusion of metastatic leiomyosarcoma to the vertebra is critical before the diagnosis of primary vertebral leiomyosarcoma can be made. There are at least 2 ways to achieve this. First, although the clinical and radiological findings resulted from the primary vertebral leiomyosarcoma are generally nonspecific, clinical and radiological examinations are still indispensable. Various imaging techniques such as x-ray, CT scan, MRI, abdominal ultrasonography, bone scan, and/or even PET/CT are recommended before the diagnosis of primary vertebral leiomyosarcoma and surgery. Second, because histological examination by HE staining cannot tell the origin of the tissue and the undifferentiated sarcoma in a case like ours, the immunohistochemical examination plays a crucial role in the diagnosis of leiomyosarcoma, especially in the differential diagnoses from other carcinomas. Immunohistochemical staining often shows that leiomyosarcoma is almost uniformly positive for both SMA and desmin, but negative for both keratins and S-100 protein.^[5]^ In addition, histological differentiation between leiomyoma and leiomyosarcoma is also important to make the right diagnosis, especially when the patient has a previous history of benign leiomyoma. In other words, lack of a history of relevant tumors, no evidence of metastatic leiomyosarcoma or other tumors detected by the systemic examinations of various imaging modalities such as CT, MRI, and PET/CT, and the results from pathological HE staining and immunohistochemical staining, a definite diagnosis of primary leiomyosarcoma of spinal vertebra can be eventually reached.

Fewer than 100 cases of primary leiomyosarcoma of the bone around the world have been reported.^[6]^ Primary leiomyosarcoma of the spinal vertebra is extremely rare and only 11 cases have been reported in the literature.^[5,7–16]^ The characteristics of these 11 cases of primary vertebral leiomyosarcoma were summarized in the Table 1.^[5,7–16]^ Among these 11 cases, 3 were male patients and 9 were female patients with an average age of 48 years. The tumor occurred in the cervical vertebra in 2 patients, in the thoracic vertebra in 6 patients, in the lumbar vertebra in 2 patients, and in the sacral vertebra in 2 patients. Pain in the local region of the primary tumor was the main symptom in these patients.

**Table 1:**
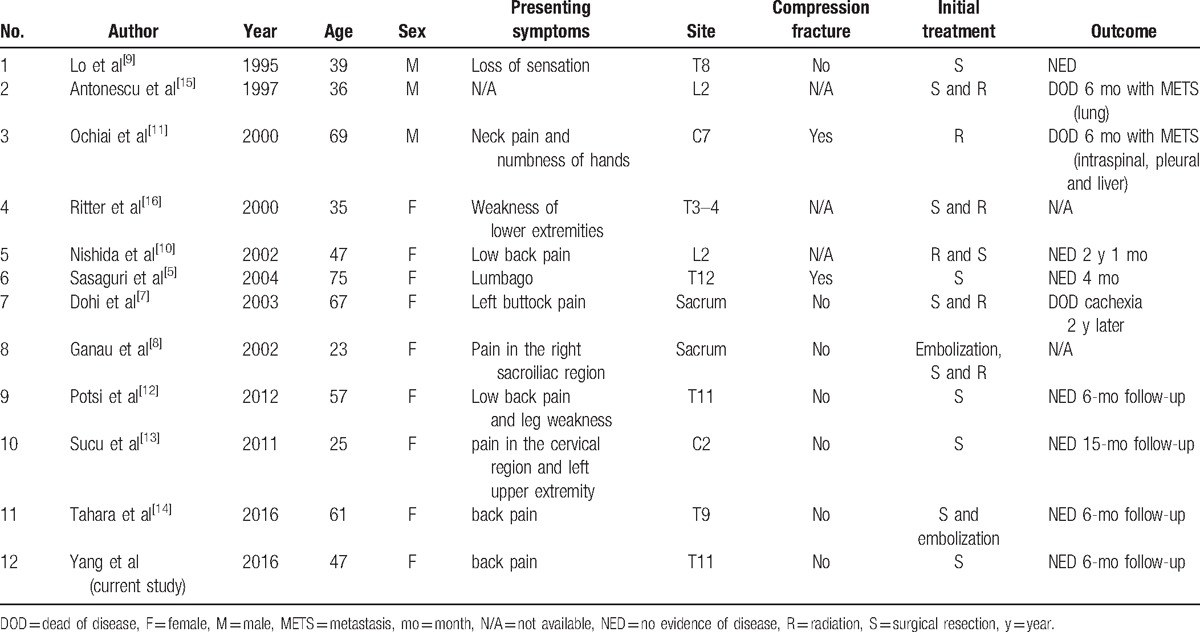
Summary of the reported cases of primary vertebral leiomyosarcoma.

Surgical resection, especially the total en bloc spondylectomy, is the main treatment option that can relieve symptoms and improve the prognosis. Radiation and embolization can also be used as alternative measures. Surgical resection with or without postoperative radiation was the main method of treatment for these 11 patients reported in the literature whose survival time was commonly >6 months. One patient was treated by radiation without surgery, 4 patients were treated by surgery without radiation, and 4 patients were treated by both surgery and radiation. Embolization was used only in 2 patients as a supplementary measure. In our patient, various imaging modalities such as x-ray, CT scan, MRI, abdominal ultrasonography, bone scan, and PET/CT were performed before surgery to help us make an accurate diagnosis and select the best treatment options. If the imaging modalities, especially the PET/CT, reported the existence of multiple metastatic tumors, the surgeons should be very careful to make a decision of surgery. In our patient, the imaging modalities showed an isolated lesion located in the T11, so surgical resection was performed to relieve symptoms and improve the prognosis. Radiation and embolization were not used in our case, considering the fact that the tumor was completely resected. Within the follow-up of 6 months, no clinical symptoms and signs of tumor recurrence were detected in our case and her clinical outcome was good, which was similar to that of the 11 cases with a limited follow-up duration previously reported in the literature.

## 4 Conclusion

Exclusion of metastatic leiomyosarcoma to spinal vertebra by various imaging modalities and histopathological examinations, especially the immunohistochemical staining with various antibodies against the epithelial and mesenchymal cell markers, are critical for the diagnosis of primary vertebral leiomyosarcoma. Surgical resection, especially the total en bloc spondylectomy, is the main choice of treatment that gives a good outcome within a limited follow-up duration.
